# Facilitating reflective learning: a PhD thesis report

**DOI:** 10.1007/s40037-012-0038-8

**Published:** 2013-01-22

**Authors:** Mirabelle Schaub-de Jong

**Affiliations:** 1 Department of Speech Therapy, School of Health Care Studies, Hanze University of Applied Sciences Groningen, 9714 CE Groningen, the Netherlands; 2 Present Address: Hoofdweg 45, 9761 EA Eelde, the Netherlands

**Keywords:** Reflection, Reflective learning, Professional behaviour, Teacher competencies, Small groups

The aim of this dissertation was to investigate conditions that may affect students’ reflective learning specifically in small groups and professional practice.

## Introduction

In medical and other health-science curricula professionalism is an important subject based on accountability for which reflective competence is an essential tool. The aim of this dissertation was to investigate conditions that may affect students’ reflective learning both in small groups and in professional practice.

## Methods

Three studies were carried out regarding the role of small-group settings, the teaching and learning methods used, and teachers’ competencies. An instrument was developed (Student perceptions of their Teachers’ competencies to Encourage Reflective Learning in small Groups: STERLinG) to assess teacher competencies in teaching reflective skills to small groups. A last study was carried out to gain an understanding of the difficulties students experience in responding to unprofessional situations in practice.

## Results

1. Students are positive about professional development courses involving reflective learning.
2. Students perceived participating in small-group settings as positive for their reflective learning.
3. The competencies of teachers of small reflective groups appear to be concerned with three domains: *supporting self*-*insight*, *creating a safe environment* and *encouraging self*-*regulation.*
4. Students had rather low scores for competencies from the domain *supporting self*-*insight* in comparison with competencies of the other domains.
5. Students recognize unprofessional situations but they do not always react to such situations.

## Discussion

Students are quite positive about professional development courses especially in small-group settings. However, to make small-group settings effective, specific conditions (such as a safe environment) have to be met. Also the role of the teacher is a key factor in facilitating reflective learning. Students were positive about their teachers’ competencies apart from competencies regarding *supporting self*-*insight.* Apparently, teachers have difficulty in teaching students to analyze the rational and emotional aspects of experiences and apply the resulting insights.

Students recognize unprofessional situations, but they have several excuses for not responding. However, students must be taught to check and discuss the validity of their excuses. At the same time, supervisors have to be aware that their behaviour may evoke excuses from the students for not reacting to (un)professional situations.

## Conclusion

Small groups are beneficial for reflective learning for which teachers need specific competencies. Teachers need to be trained in facilitating students’ reflective learning. To further develop the reflective competence of students during clinical practice it is important to discuss unprofessional situations in small groups. Students should learn to check the validity of excuses for not responding. Clinical supervisors play an important role in this process.

